# The Effect of High-Pressure Homogenization Conditions on the Physicochemical Properties and Stability of Designed Fluconazole-Loaded Ocular Nanoemulsions

**DOI:** 10.3390/pharmaceutics16010011

**Published:** 2023-12-20

**Authors:** Agnieszka Gawin-Mikołajewicz, Urszula Nawrot, Katarzyna Hanna Malec, Karolina Krajewska, Karol Przemysław Nartowski, Bożena Lucyna Karolewicz

**Affiliations:** 1 Department of Drug Form Technology, Wroclaw Medical University, 211A Borowska Str., 50-556 Wroclaw, Poland; k.malec@umw.edu.pl (K.H.M.); karolina.krajewska@student.umw.edu.pl (K.K.); karol.nartowski@umw.edu.pl (K.P.N.); bozena.karolewicz@umw.edu.pl (B.L.K.); 2 Department of Pharmaceutical Microbiology and Parasitology, Wroclaw Medical University, 211A Borowska Str., 50-556 Wroclaw, Poland; urszula.nawrot@umw.edu.pl

**Keywords:** drug delivery, surfactants, ocular nanoemulsion, high-energy methods, process parameters, formulation optimization, fluconazole, stability examined via NMR

## Abstract

The growing interest in high-energy emulsification is a result of its scalability, which is important from an industrial perspective and allows for a more reproducible and efficient production of pharmaceutical formulations. The aim of this study was to evaluate the effect of composition, mainly a fixed surfactant/cosurfactant (S_mix_) ratio, their concentration, and the parameters of high-pressure homogenization (HPH) processing on the quality and stability of ophthalmic fluconazole-loaded nanoemulsions. After a physicochemical analysis of nanoemulsions containing 20% *w*/*w* of oil, as optimal conditions for the HPH process, three cycles at a pressure of 1000 bar were established, obtaining formulations with an average droplet diameter size in the range of 80.63–129.68 nm and PDI values below 0.25. While it was expected that an increasing cosurfactant concentration decreased the droplet size, in the case of formulations containing Tween 20 and 10% *w*/*w* of cosurfactants, “over-processing” was observed, identified by the droplet size and polydispersity index increase. Consecutively, the selected formulations were evaluated for in vitro drug release in Franz’s cell, antifungal activity, and 30-day stability using NMR spectroscopy. An antifungal activity test showed no significant difference in the antifungal activity between optimal fluconazole-loaded nanoemulsions and a 0.3% aqueous drug solution, but previously, research showed that prepared formulations were characterized by a higher viscosity and satisfactory prolonged release compared to a control. In a 30-day stability study, it was observed that higher HLB values of the used surfactants decreased the stability of the formulations in the following order: Kolliphor EL, Tween 80, Tween 20. The NMR spectra confirmed that Kolliphor EL-based formulations ensured the higher stability of the nanoemulsion composition in comparison to Tween 80 and a better stabilizing effect of propylene glycol as a cosurfactant in comparison to PEG 200. Therefore, the optimization of HPH technology should be focused on the selection of S_mix_ and the S_mix_:oil ratio in order to prepare stable formulations of high quality.

## 1. Introduction

Nanoemulsion development is considered an interesting and promising research direction to improve the delivery of ophthalmic drugs [1,2,3,4]. Despite their confirmed therapeutic benefits, the formulation of these innovative ocular carriers poses a challenge because of their multicomponent nature and the extensive optimization required to obtain a product with appropriate properties. In the preparation of nanoemulsions, the selection of suitable technological processes is of great importance to obtain a formulation with guaranteed stability [5,6]. To reduce the size of the internal oil phase to a nanometric range, high-energy methods, e.g., high-pressure homogenization (HPH) [7] and microfluidization [8], are generally preferred for the industrial production of nanoemulsion systems [6,9,10]. Because of its high efficiency, scalability, process repeatability, customizable droplet size, and production scale-up, HPH is a preferred technique [9,11,12,13]. Moreover, all processing steps of molecular liquids in HPH, including the impact of the cavitation, turbulent, and shear forces, allow for the achievement of consistent, uniform particle sizes with a favorable low polydispersity index [14,15]. Although significant progress has been made in the optimization of high-pressure homogenization processes, there is still a limited number of reports on the effects of homogenization parameters. The recognized parameters, like the number of cycles and homogenization pressure, influence the feasibility of ocular nanoemulsion formulation and their properties, such as an inner phase particle size [12], surface charge, and polydispersity index, which, altogether, affect the stability of these formulations [15]. In the homogenization process, the low polydispersity of a formulation is usually provided by increasing the number of homogenization cycles [16,17]. However, an increased number of cycles can reduce the efficiency of the process and affect the quality of the product due to the generation of large heat amounts, which is a limitation of HPH technology [18,19,20]. In the literature concerning ocular formulation technology, a scarce number of studies include implementing high-pressure homogenizers to prepare nanoemulsions. There are reports regarding the optimization of nanoemulsion preparation in the HPH process for timolol [21], cyclosporine A [22,23], tacrolimus [7], and rifampicin [24]. Gallarate et al. processed a pre-prepared macroemulsion with timolol using HPH with three cycles of 5 min at a pressure of 1000 bar. As a result of the homogenization process, stable, biocompatible, sterilizable, and non-irritating nanoemulsions were obtained [21]. Lallemand et al. developed a Novasorb cationic nanoemulsion with cyclosporine A to treat dry eye syndrome. For this purpose, the pre-emulsion with a size of oil droplets of about 1 µm was processed using the HPH method at 1000 bar and under a temperature of 4 °C, finally resulting in an internal phase droplet size in the range of 150–200 nm [22,23]. To date, based on Novasorb technology, Novagali Pharma has successfully launched two products with cyclosporine A on the market, i.e., Cyclokat^®^, for the treatment of dry eye, and Vekacia^®^, which is indicated in vernal keratoconjunctivitis [22]. Additionally, Henostroza et al. prepared rifampicin nanoemulsions from pre-emulsion using HPH at 10,000 psi (690 bar) in five cycles, obtaining an inner phase particle size of about 150 nm. The presented formulations demonstrated a homogenous appearance and a monomodal distribution of droplet size over 90 days in a stability test. An in vitro antibacterial activity evaluation of the anionic nanoemulsion with rifampicin that was further processed into the cationic nanoemulsion via chitosan and polymyxin B addition showed growth inhibition of *Mycobacterium tuberculosis* similar to the standard rifampicin suspension (10 μg/mL), being a positive control. No adverse effect of the HPH process and surface modification process on the antibiotic activity was observed. The authors showed that the obtained nanoemulsions may enable a promising alternative for ophthalmic products in the treatment of ocular tuberculosis, with potentially longer intervals between administrations based on the presented mucoadhesion evaluation [24].

Fluconazole (FLZ) is a substance that is slightly soluble in water (in an octanol/aqueous buffer with a pH of 7.4; the log P value of FLZ is 1.0, with an aqueous solubility of 5.5 mg/mL), and its hydrophobic nature presents a constant challenge in developing a suitable ophthalmic dosage form [25]. There are some research reports on new ophthalmic formulations with FLZ, such as nanoemulsified in situ gels obtained using Campul MCM, Tween 80, Transcutol P, and Carbopol 934 (oil phase, surfactant, cosurfactant, and gelling agent, respectively) [25]; microemulsions containing isopropyl myristate with a 3:1 mixture of Tween 80 and PEG 400 (oil phase, surfactant, and cosurfactant, respectively); and niosomal gels based on Carbopol 934 as a gelling agent with Span 60 and cholesterol [26]. None of these formulations were obtained via high-energy and scalable technology, which is important from an industrial perspective. The aforementioned nanoemulsions with FLZ obtained by Pathak et al. were prepared via the low-energy spontaneous emulsification method, using a 20.25% content of the surfactant and only 7% of the oil phase [25]. Accordingly, microemulsions prepared by Soliman et al. contained high concentrations (30–40%) of the surfactant/cosurfactant mixture and 45–60% of the oil phase, resulting in a high viscosity value, which can cause difficulties with product instillation, eye irritation, and reflex tearing [26,27]. Soliman et al. prepared niosomal gels with FLZ in a multi-step process by adding an aqueous dispersion of niosomes into the gel base [26]. The raw niosomal dispersions have limited stability, and the size of niosomes often increases during storage, thus changing the efficiency of the substance encapsulation. This makes the final formulation with niosomes less stable and requires parameter control at all steps, resulting in a time-consuming technology process [26,28,29]. Nevertheless, new carriers (i.e., a gel containing niosomes or microemulsions) have achieved a significantly sustained release of FLZ, higher bioavailability, and lower drug elimination rates in various tissues of the eye and the aqueous humor compared to marketed formulations, which indicates a beneficial potential of oil-based formulations in the development of novel ophthalmic dosage forms containing fluconazole [26].

The objective of our study was to evaluate the feasibility of nanoemulsion preparation with FLZ using a high-pressure homogenization technique. The effect of composition, mainly a fixed surfactant/cosurfactant (S_mix_) ratio, their concentration (15–20%), and the process parameters (i.e., the number of cycles, pressure, temperature, and the applied membrane sterilization method) on the physicochemical and pharmaceutical characteristics of nanoemulsions, including the particle size and polydispersity index, was evaluated. Furthermore, to the best of our knowledge, NMR spectroscopy was involved, for the first time, to investigate changes at the molecular level occurring upon storage of the selected nanoemulsions.

## 2. Materials and Methods

### 2.1. Materials

Fluconazole (FLZ) was received as a gift from Hasco-Lek S.A. (Wroclaw, Poland). Isopropyl myristate, castor oil, olive oil, peanut oil, cottonseed oil, sesame oil, Pluronic F127 (PLU), Tween 80 (T80), Tween 20 (T20), Kolliphor EL (KOL), polyethylene glycol 200 (PEG 200), and Span 80 were purchased from Sigma-Aldrich Chemical Company (St. Louis, MO, USA). Oleic acid (OA) and propylene glycol (PG) were obtained from Chempur (Piekary Slaskie, Poland). High-performance liquid chromatography (HPLC) grade methanol was purchased from Honeywell Specialty Chemicals (Seelze, Germany). Deuterium oxide was purchased from Armar Chemicals (Leipzig, Germany). All chemicals used in this study were of analytical grade.

Four *Candida* strains were involved in this study from American Type Culture Collection (Manassas, VA, USA) from the laboratory collection of the Department of Pharmaceutical Microbiology and Parasitology at the Wroclaw Medical University (*C. albicans* ATCC 90028, *C. parapsilosis* ATCC 90018, *C. glabrata* ATCC 90030, *C. tropicalis* ATCC 750). The strains were stored in trypticase soy broth with the addition of 15% glycerol and kept at −80 °C. 

### 2.2. Screening of Oils, Surfactants, and Cosurfactants as the Components of the Fluconazole-Loaded Nanoemulsion

Selection of the components is extremely important, as it provides good solubilizing efficiency of the drug, which is essential for developing a nanoemulsion. Hence, the solubility of FLZ in the selected oils, surfactants, cosurfactants, and water was determined. The drug was added in excess into 2 mL of each excipient or water, and the samples were shaken using a vortex (Heidolph Instruments, Schwabach, Germany) at 100 rpm for 2 min. Then, the samples were kept and shaken in a thermostatic shaking water bath (Memmert, Schwabach, Germany) at 25 °C for 48 h to obtain the equilibrium. The samples obtained were centrifuged at 5000 rpm for 10 min (MPW-52 Med. Instruments, Warsaw, Poland) and filtrated through 0.45 μm PVDF membrane filters (Macherey-Nagel, Düren, Germany) to remove the undissolved FLZ. Then, 200 μL of the supernatant was diluted with a mixture of water and methanol 50:50 *v*/*v* to 10 mL, filtrated through a 0.20 μm PTFE membrane filter (Merck, Darmstadt, Germany), and the FLZ content was determined using the HPLC method described in Section 2.3. Each experiment was performed in triplicate.

### 2.3. The Quantitative Determination of the FLZ Content via High-Performance Liquid Chromatography (HPLC)

The quantitative determination of the FLZ content was performed using an HPLC Agilent 1260 Infinity with UV-DAD detection (Agilent Technologies, Inc., Santa Clara, CA, USA). The mobile phase consisting of a mixture of water and methanol 60:40 *v*/*v* was delivered in an isocratic elution. The flow rate was set to 1.0 mL/min through a Zorbax SB-C18 (150 mm × 4.6 mm, 5 µm) column (Agilent Technologies, Inc., Santa Clara, CA, USA). The column oven temperature was maintained at 30 °C. FLZ was detected at a wavelength of 211 nm, with a retention time of 4.4 min. The concentration of FLZ was calculated using a calibration curve constructed in a concentration range of 7.64–191.00 µg/mL, with a mean correlation coefficient of ≥0.9999.

### 2.4. Optimization of the High-Pressure Homogenization Parameters in the Preparation of the Fluconazole-Loaded Nanoemulsion

#### 2.4.1. Selection of the Optimal Homogenization Pressure

To select the optimal homogenization pressure, three selected fluconazole-loaded emulsion formulations with Kolliphor (NE1_KOL), Tween 20 (NE6_T20), and Tween 80 (NE11_T80) were prepared under different homogenization pressures: 500, 1000, 1500 bar (applying three cycles) (Table 1). Firstly, FLZ with oleic acid was mixed for 8 h at 25 °C, using a magnetic stirrer (IKA Industrie, Königswinter, Germany) at 200 rpm; then, the surfactant was added to the mixture and stirred for 16 h at 200 rpm. Subsequently, an appropriate amount of water was added and mixed for 1 h at 200 rpm. Each coarse emulsion was homogenized using a Panda Plus 2000 high-pressure homogenizer (GEA Mechanical Equipment, Parma, Italy). After each cycle, the samples were cooled down to room temperature to prevent the temperature from affecting the particle size. Based on the determined droplet size distribution and polydispersity index for the abovementioned homogenized formulations, the most suitable homogenization pressure, namely, 1000 bar, was chosen for further studies.

#### 2.4.2. Selection of the Number of Passes under the Optimal Homogenization Pressure

**Figure 1:**
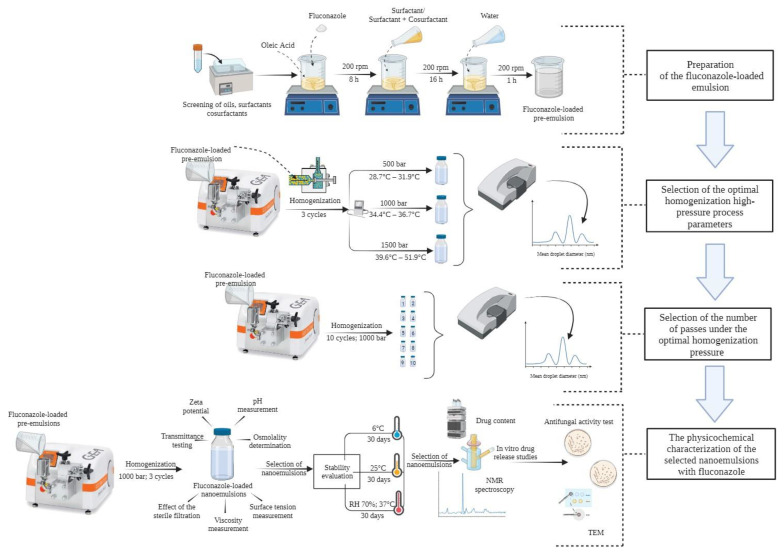
Schematic representation of the optimization of the HPH parameters in the preparation of fluconazole-loaded nanoemulsions and the physicochemical characterization of the prepared formulations.

Emulsions were prepared according to the procedure described in Section 2.4.1, viz. FLZ with oleic acid and mixed for 8 h at 25 °C using a magnetic stirrer (IKA Industrie, Königswinter, Germany) at 200 rpm; then, the surfactant and cosurfactant were added to the mixture and stirred for 16 h at 200 rpm. Subsequently, an appropriate amount of water was added and mixed for 1 h at 200 rpm. Afterward, each coarse emulsion, prepared according to Table 1, was passed 1–10 times through the homogenizer, under the optimum homogenization pressure of 1000 bar, and then, the droplet size and polydispersity index evolution after each pass were investigated (Figure 1). After evaluating the properties of the nanoemulsion formulation, three passes at a pressure of 1000 bar were considered optimal for processing in the high-pressure homogenizer.

#### 2.4.3. Measurement of the Particle Size (PS) and Polydispersity Index (PDI)

The average particle size, droplet size distribution by intensity, and polydispersity index of nanoemulsion formulations were determined via the dynamic light scattering (DLS) technique using a Zetasizer Nano ZS ZEN3600 (Malvern Instruments Ltd., Worcestershire, UK), fitted with a 4 mW He-Ne laser operating at 633 nm. The Non-Invasive Back-Scatter (NIBS) method was used at a scattering angle of 173°. Each formulation (0.2 mL) was diluted with deionized water (9.80 mL) to prevent a multiple scattering effect and measured at 25 °C. The results are expressed as the mean and standard deviation of three independent measurements. The duration and set position of each measurement were fixed automatically via the apparatus.

### 2.5. Effect of Sterile Filtration on the PS and PDI Parameters of the Nanoemulsions 

To evaluate the influence of the filtration method on the physicochemical properties of nanoemulsions, the prepared formulations, after the third cycle of homogenization under an optimum homogenization pressure of 1000 bar, were filtrated through pyrogen-free MCE membrane filters with a pore size of 0.22 µm (Filtrakon, Laziska Gorne, Poland). Each filtered formulation (0.2 mL) was diluted with deionized water (9.80 mL). Then, the droplet size and polydispersity index were determined via photon correlation spectroscopy, using a Zetasizer Nano ZS ZEN3600 (Malvern Instruments Ltd., Worcestershire, UK).

### 2.6. Physicochemical Characterization of the Nanoemulsions Prepared Using the Optimal Homogenization Parameters

#### 2.6.1. Visual Examination and Transmittance (%T) Testing

The prepared formulations were examined visually under a light source against an alternating white and black background. The possible occurrence of phase separation, color changes, and sedimentation was evaluated straight after the preparation and 2 h later. The percentage transmittance (%T) of the nanoemulsions diluted 10 and 500 times in deionized water was measured in triplicate using a UV/VIS spectrophotometer Jasco V—650 (Jasco International CO., Ltd., Tokyo, Japan) at a wavelength of 630 nm.

#### 2.6.2. Refractive Index Measurement

The refractive index was determined in triplicate at 21 °C using a DR 201-95 refractometer (A. Krüss Optronic GmbH, Hamburg, Germany).

#### 2.6.3. Measurement of the Zeta Potential (ZP)

The zeta potential of formulations was determined via photon correlation spectroscopy using a Zetasizer Nano ZS ZEN3600 (Malvern Instruments Ltd., Worcestershire, UK). Light scattering was monitored at 25 °C at a measurement angle of 173°. The dispersed formulations were measured in triplicate after the dilution in deionized water (1:50 *v*/*v)*.

#### 2.6.4. pH Measurement

Measurements of pH values were performed in triplicate potentiometrically at 25 °C using a CPC-511 pH-meter (Elmetron, Zabrze, Poland) with an electrode InLab^®^ Flex-Micro (Mettler Toledo, Warsaw, Poland).

#### 2.6.5. Osmolality Determination

The osmotic pressure was measured in triplicate for all formulations at 25 °C using an Osmometer Os 3000 (Marcel S.A., Zielonka, Poland).

#### 2.6.6. Surface Tension Measurement

Surface tension measurements were carried out in triplicate at 25 °C using a Sigma 703 tensiometer (KSV Instruments Ltd., Helsinki, Finland) provided with a Du Nouy ring (the ring radius was 9.545 mm, while the wire radius was 0.185 mm).

#### 2.6.7. Viscosity Determination

The viscosity of the samples (0.5 mL) was measured in triplicate at 25 °C ± 0.5 °C and 34 °C ± 0.5 °C with a speed rate of 100 rpm and a shear rate 750 s^−1^ using a Brookfield RV DV III+ Digital Rheometer (Brookfield Engineering Laboratories, Inc., Middleboro, MA, USA) with a Brookfield CPE 40 spindle.

### 2.7. Stability Evaluation

To evaluate the stability of the selected nanoemulsions, samples were kept in glass vials at refrigerator temperature (6 ± 0.5 °C), room temperature (25 ± 1 °C), and under accelerated stability testing conditions (37 ± 2 °C/RH 70 ± 5%). The formulations were evaluated for physical instability issues, including phase separation, coalescence, drug sedimentation, and changes in the particle size and index polydispersity at the predetermined time intervals, up to 30 days [30,31,32].

### 2.8. Evaluation of the Properties of the Nanoemulsions Selected in the Stability Test

#### 2.8.1. Nuclear Magnetic Resonance (NMR) Spectroscopy Assay

The NMR spectra were acquired using a Bruker NMR Avance III spectrometer operating at 600.13 MHz for ^1^H and equipped with a broadband SmartProbe. Presaturated ^1^H NMR and ^1^H-^1^H NOESY NMR (at mixing time 0.3 s) spectra were recorded for nanoemulsions loaded with FLZ (freshly prepared—series 1 and after a 30-day stability test at room temperature—series 2), control samples without the drug (blank series), and oil solution of FLZ in oleic acid (F_OA). The selected formulations for the examination passed the stability tests at room temperature after 10, 20, and 30 days, not showing any signs of sedimentation, phase separation, or delamination. A quantity of 200 µL of each sample was transferred to 5 mm borosilicate glass test NMR tubes with an insert with acetone-d_6_ introduced. Chemical shifts were assigned based on the published data [33,34,35,36,37,38].

#### 2.8.2. Drug Content

A volume of 100 μL of the fluconazole-loaded nanoemulsions selected in the 30-day stability test was diluted with a mixture of water and methanol at 50:50 *v*/*v* up to 10 mL, stirred for 2 h at 200 rpm using a magnetic stirrer (IKA Industrie, Königswinter, Germany), and drug content was determined via the HPLC method described in Section 2.3.

#### 2.8.3. In Vitro Release Studies

In vitro release studies were performed using a Vision Microette Automated Diffusion Test System (Hanson Research Corp., Chatsworth, CA, USA) consisting of a Vision Microette autosampler, a 6-cell Drive System with 6 vertical diffusion cells (VDCs) (modified Franz’s cells), a programmable circulating water bath, and a Vision AutoFill sample collector. Franz’s cells with an effective surface of 1.77 cm^2^ were filled with 7 mL of phosphate buffer (pH 7.4) and stirred at 200 rpm, while the temperature was maintained at 37 ± 0.5 °C. Subsequently, 0.5 mL of the selected fluconazole-loaded nanoemulsion or 0.5 mL of the 0.3% drug solution (being the control sample) was applied on a previously soaked dialyzing membrane (MWCO 12–14 kDa) in Spectra/Por^®^ Dialysis Membrane Standard Discs (Spectrum Laboratories Inc., Rancho Dominguez, CA, USA) in Franz’s cells. A volume of 0.5 mL of the samples was collected at regular intervals (0.25, 0.5, 1, 2, 3, 4, 5, 6, 8, 10, 12, 16, 24 h) and replaced with the same volume of the fresh media. The samples were subsequently diluted with methanol 50:50 *v*/*v* and analyzed for their FLZ content via the HPLC method described in Section 2.3.

#### 2.8.4. Surface Morphology—Transmission Electron Microscopy (TEM)

The structure and morphology of the nanoemulsions were studied using a JEOL 1200 EX transmission electron microscope (JEOL Ltd., Tokyo, Japan) at an accelerating voltage of 80 kV. A volume of 10 µL of diluted sample solution (1:100) was placed on a copper mesh (400 grids) with a film and carbon coating (Agar Scientific, Stansted, UK). The sample was left for 5 min and carefully blotted with filter paper. Subsequently, 4 µL of 2% uranyl acetate (MicroShop, Piaseczno, Poland) was used for negative staining. After 60 s of staining, the grid was blotted from the top and allowed to dry in normal conditions for at least 1 h. A TVIPS TemCam-XF416 camera (TVIPS GmbH, Gauting, Germany) was used to determine the shape of the dispersed phase.

#### 2.8.5. Antifungal Activity Test

The in vitro antifungal activity of the optimized fluconazole-loaded nanoemulsions and the FLZ solution (0.3% *w*/*w*) as the reference was measured via disk diffusion method using representative *Candida* strains, as described in Section 2.1 [39]. The studied fungi were cultured on the Sabouraud dextrose agar plates (BioMaxina, Lublin, Poland) for 24 h at 35 °C, and then, the suspension of each strain was prepared in 0.9% NaCl at a concentration of 0.5 McFarland. The suspensions of the yeasts were spread with a sterile swab on the Mueller–Hinton agar supplemented with 2% dextrose and 0.5 µg/mL methylene blue (BioMaxima, Lublin, Poland). Subsequently, 6 mm diameter paper discs (BioMaxima, Lublin, Poland) were soaked with 10 µL of the prepared fluconazole-loaded nanoemulsions, blank formulations, and the 0.3% drug solution and were placed on plates. After 24 h incubation at 35 °C, the plates were observed for an antifungal inhibition zone around the disks, which was measured using a millimeter ruler. The experiment was repeated three times.

### 2.9. Statistical Analysis

Data concerning the particle size of the dispersed phase before and after filtration were statistically verified using Student’s *t*-test for the independent samples and the non-parametric Mann–Whitney U test. All statistical analyses were performed using Statistica 13.3. *p*-Values <0.05 were considered statistically significant. The results of the antifungal activity test were compared using the non-parametric Kruskal–Wallis test with Dunn’s multiple comparison test using GraphPad Prism (Version 8.0.1; GraphPad Software Inc., La Jolla, CA, USA).

## 3. Results and Discussion

### 3.1. Screening of Oils, Surfactants, and Cosurfactants as the Components of the Fluconazole-Loaded Nanoemulsion

Preliminary studies were carried out to select ingredients for the feasibility of the fluconazole-loaded nanoemulsion. The selection of the most suitable oil, surfactant, and cosurfactant is extremely important as it provides good solubilizing efficiency of the drug [40,41], which is essential for the nanoemulsion preparation and ingredients’ safety in a given concentration after ocular application [3,42]. For preliminary FLZ solubility studies, the vegetable oils, long-chain fatty acid ester (isopropyl myristate), and long-chain unsaturated fatty acid (oleic acid) well tolerated by the eye were selected [43,44,45,46]. The results of the solubility of FLZ in the selected oils, surfactants, cosurfactants, and water are presented in the Supplementary Information (SI) in Table S1. After the dissolution screening test for the internal phase components, OA (FLZ solubility ~13.42 ± 0.01 mg/mL) was selected for the nanoemulsion formulation loaded with FLZ. OA is a potential enhancer of the penetration of drugs through the corneal lipophilic barriers, increasing the fluidity of intercellular lipid barriers by creating discrete domains that disrupt the continuity of the corneal multilayer epithelium and induce highly permeable pathways [47,48]. Moghimipour et al. used OA to prepare celecoxib-loaded ophthalmic nanoemulsions with oil phase concentrations of 5% and 50%, containing OA and Transcutol P in a ratio of 10:1. They obtained formulations with much higher coefficients of drug permeability parameters through the rabbit cornea [49].

When selecting a surfactant that will ensure a very low interfacial tension and prevent the coalescence of oil droplets during homogenization, the percentage of the oil phase content, the type of emulsifier, and the HLB value should be considered [7,19]. According to the literature, to obtain a 20 wt % o/w nanoemulsion, a 5–10 wt % surfactant concentration is required [50]. In this study, to prepare o/w ophthalmic nanoemulsions, nonionic surfactants with HLB values above 10 were used [51,52]. These surfactants are the representatives of the main type of surface-active agents used in ophthalmology due to their advantages with respect to compatibility, stability, and non-toxicity compared to their cationic, anionic, or amphoteric counterparts [53]. Tween 20 and 80 have been used as examples of nonionic surfactants classified as practically non-irritant, applied in a wide range of concentrations (up to 100% *w*/*w*) and as surfactants in many commercial ophthalmic formulations [19,52,54,55,56]. Additionally, due to the longer hydrocarbon chain length in T80 in comparison to T20, the former can broaden the area of the created nanoemulsion region [57,58]. Other examined components, namely, Kolliphor EL (macrogolglycerol ricinoleate) and Pluronic F127 (triblock PEO−PPO−PEO copolymers of poly(ethylene oxide) (PEO) and poly(propylene oxide) (PPO)), have also been added to ophthalmic formulations as low-toxicity nonionic surfactants [59,60]. Data from the literature indicate that the application of a 30% aqueous solution of Kolliphor EL [44] and 20% of Pluronic F127 had a non-irritant effect on the eyeball [60]. Furthermore, Pluronic F127 is used to increase the viscosity of eye drops, which leads to increased corneal contact time and, consequently, drug bioavailability [61]. 

To prepare stable nanoemulsion formulations, cosurfactants that can increase the miscibility of both phases and ensure liquid–liquid interface fluidity are also used [62]. As cosurfactants for the designed fluconazole-loaded nanoemulsions, PG, PEG 200, and Span 80 (sorbitan monooleate) in concentrations up to 10% were selected. Amongst all studied cosurfactants, PG exhibited the greatest solubility of FLZ (25.60 ± 0.03 mg/mL). PG, according to the literature, does not irritate rabbit eyes, even after the application of a 50% solution [44,63]. In the case of PEG 200, Gallarte et al. showed a non-irritating effect on rabbit eyes using a prepared o/w microemulsion with levobunolol in a 19.5% aqueous solution of PEG 200 [64].

Based on the FLZ solubility screening, 20% *w*/*w* OA was finally selected as the internal phase component for the further development and optimization of drug-loaded nanoemulsion formulations.

### 3.2. Optimization of the High-Pressure Homogenization Parameters in the Preparation of the Fluconazole-Loaded Nanoemulsion

**Figure 2:**
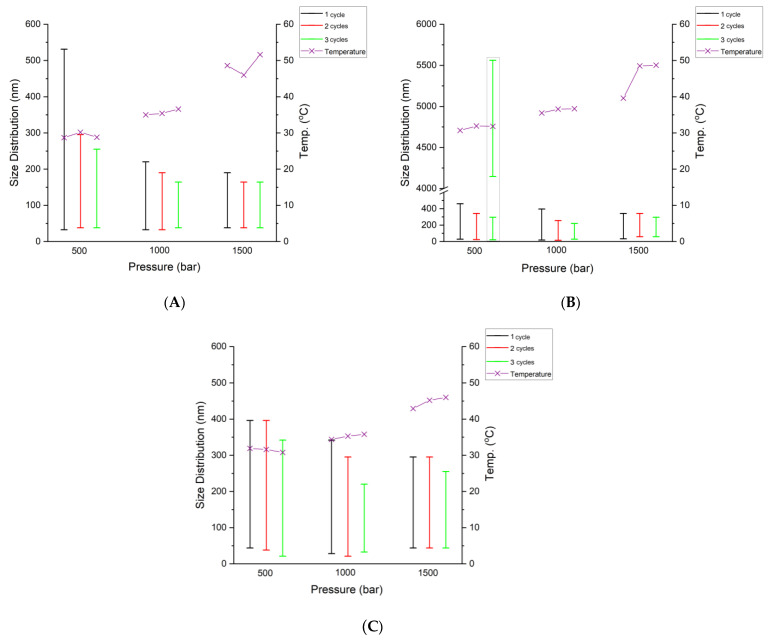
Effect of pressure (500, 1000, or 1500 bar) and number of passes (from 1 to 3) involved in the homogenization process on the particle size distribution by intensity and outlet temperature of the prepared formulations. (**A**) NE1_KOL; (**B**) NE6_T20; (**C**) NE11_T80.

In the HPH method used for the nanoemulsion preparation, both the number of passes and pressure affect the properties and stability of the produced nanoemulsion [65]. The optimization of the nanoemulsion preparation parameters using HPH technology aims at an efficient production of nanoemulsions with the lowest possible number of passes, pressure, and controlled outlet temperature. All of these features are important for the stability of the substance in the formulation [66]. In order to select the pressure of the HPH process, the effects of the homogenization pressures (500, 1000, and 1500 bar) and an initial three passes through the homogenizer on the average droplet size distribution and outlet temperature after the homogenization process were studied for three fluconazole-loaded emulsions containing Kolliphor (NE1_KOL, Figure 2A), Tween 20 (NE6_T20, Figure 2B), and Tween 80 (NE11_T80, Figure 2C). Using a process pressure of 500 bar, the outlet temperature of the prepared formulations after three passes was obtained in the range of 28.8–31.8 °C, with a wide particle size distribution (21.04–5560 nm), and in the case of the NE6_T20 formulation with Tween 20, with a bimodal profile of the droplet size distribution (first peak, 21.04–295.3 nm; second peak, 4145–5560 nm). When a pressure of 1500 bar was applied to the homogenization process, the outlet temperature of the prepared formulations increased to a range of 39.6–51.9 °C after 1-3 passes. In addition, after three passes through the homogenizer, the particle size distribution of the dispersed oil phase increased for the NE6_T20 and NE11_T80 formulations (43.82–295.3 nm), compared to the process pressure of 1000 bar (28.21–220.2 nm). When all the abovementioned factors were taken into account, a pressure of 1000 bar was considered the optimal emulsion processing condition. This allowed for obtaining a unimodal particle size distribution with a particle size reduction during the successive passes and an outlet temperature of the formulations up to 35.0–36.6 °C.

**Figure 3:**
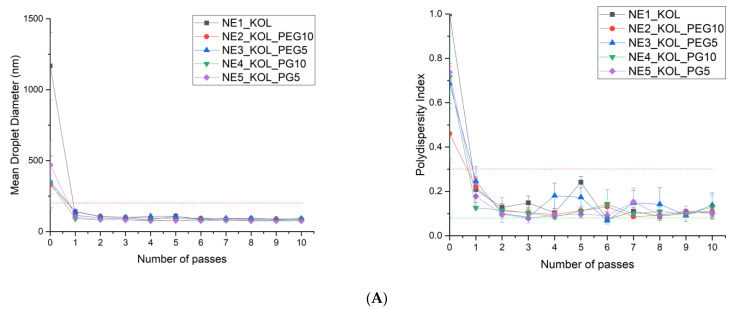

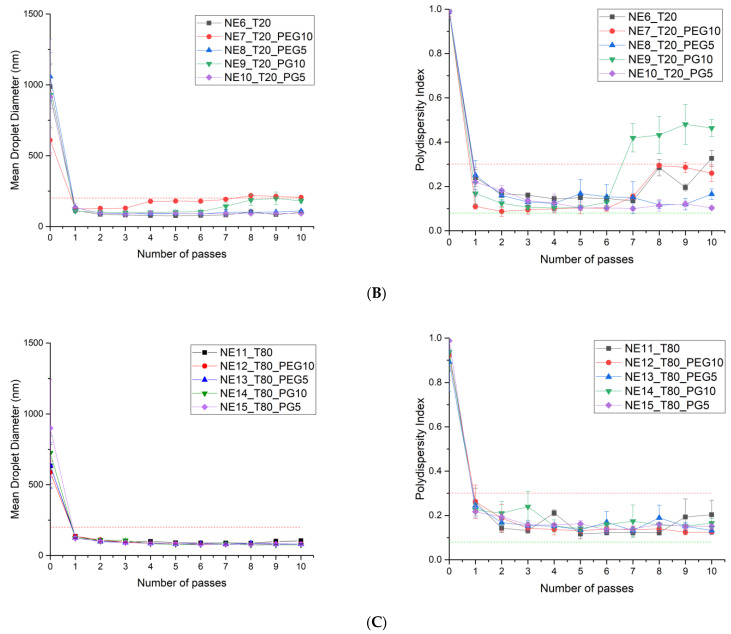
Effect of the number of homogenization passes (from 0 to 10) at 1000 bar on the mean droplet diameter and polydispersity index of the prepared fluconazole-loaded nanoemulsions. (**A**) with KOL; (**B**) with T20; (**C**) with T80 as surfactants.

In a further study, the effect of the number of passes through the high-pressure homogenizer at an optimized pressure of 1000 bar on the droplet size distribution and PDI was investigated. Thus, 18 coarse emulsions containing 0.3% *w*/*w* FLZ were prepared, according to the compositions in Table 1, and all were passed through the homogenizer 1–10 times at a pressure of 1000 bar (Figure 3). All coarse emulsions displayed a very broad, polymodal particle size distribution with PDI values equal to 1.00. After the first pass through the homogenizer, a significant reduction in the average droplet size of 77.01–90.15% was observed, as well as a reduction in PDI values of 51.87–88.92% for all formulations, compared to coarse emulsions. However, the particle size distribution of the prepared emulsions was still bimodal with the peak maxima at 91.28–141.80 nm and 4145–5560 nm. After two passes through the homogenizer, we observed a subsequent reduction in the mean droplet size and a decrease in the PDI values, compared to the formulations after one cycle of homogenization. A second fraction of the large particles above 4000 nm, in the case of the formulations NE8_T20_PEG5, NE10_T20_PG5, NE11_T80, NE14_T80_PG10, and NE15_T80_PG_5, was also observed. An example of the average size distribution curves as a function of the number of passes at 1000 bar for the NE15_T80_PG5 formulation is shown in the SI (Figure S1). After only three passes through the homogenizer, the droplet size distribution proved to be monomodal for all nanoemulsions, with the average droplet diameters in the range of 80.63–129.68 nm and PDI values below 0.25. The data from the literature indicate that PDI values for monodisperse samples below 0.2 confirm a narrow size distribution [67,68], which provides good long-term formulation stability due to the reduced degradation processes, such as Ostwald ripening [25]. A further increase in the number of passes did not lead to a decrease in the droplet size and changes in PDI values, whereas it resulted in an increase in the droplet size and PDI values above 0.3 for formulations containing T20 as the surfactant and a 10% addition of cosurfactants, i.e., NE7_T20_PEG10 and NE9_T20_PG10. It has been reported in the literature that an increasing number of passes in the HPH method can cause an increase in droplet size. Probably, this is a consequence of the too-low concentration and adsorption rate of the emulsifier needed to cover the increasing oil–water interface during prolonged homogenization or the temperature increase due to the multiple passes through the homogenizer, thus resulting in coalescence [69,70].

Based on the obtained results regarding the mean particle size distribution and PDI, further experiments were limited to three HPH cycles at a pressure of 1000 bar. The formulations containing Pluronic F127 (NE16_PLU, NE17_PLU_PEG10, and NE18_PLU_PG10) as a surfactant were eliminated from further studies due to the gelation of the samples, which made it difficult to measure the size of the dispersed phase using the DLS method.

### 3.3. Effect of Sterile Filtration on the Mean PS and PDI

The main requirement for ophthalmic formulations is sterility; therefore, filtration has been widely adopted as a sterilization method during the preparation of nanoemulsion formulations [3,71,72]. However, the possible effect of filtration on the physicochemical properties of the formulation and drug concentration in the product should be taken into account [72,73]. In our study, the effect of filtration through 0.22 µm filters made of mixed cellulose esters on the particle size of the dispersed phase of the prepared nanoemulsion was analyzed after three passes through the homogenizer (SI, Figure S2). Slight but statistically significant changes (*p* > 0.05) in the mean droplet size were observed for the NE11_T80 and NE14_T80_PG10 samples and formulations containing T20, with the exception of the NE10_T20_PG5 nanoemulsion. Depending on the composition, sterile filtration resulted in a statistically significant increase in the droplet size of 1.94–3.69%. On the contrary, a decrease in the droplet size of 1.04–5.69% was registered for the NE9_T20_PG10, NE11_T80, and NE14_T80_PG10 formulations. Moreover, sterile filtration resulted in statistically significant differences in the PDI values, namely, a 6.64–23.30% decrease for the NE6_T20, NE7_T20_PEG10, and NE13_T80_PEG5 nanoemulsions and a 40.22% increase for the NE9_T20_PG10 nanoemulsion. In all nanoemulsions containing KOL as a surfactant, sterile filtration showed no significant differences in the mean nanodroplet size and PDI values between initial and post-filtration results, suggesting that filtration may be suitable for the sterilization of these nanoemulsions. Gue et al. sterilized the prepared nanoemulsions using 0.2 µm regenerated cellulose syringe filters and assessed that filtration did not affect their properties, such as the average particle diameter (3.41% decrease) and PDI values (15.88% increase), as well as ZP, pH, and osmolarity [71]. Ismail et al. also showed that the filtration of a travoprost-loaded nanoemulsion through 0.22 µm cellulose filters did not affect any of the following parameters: PS (0.38% decrease), PDI (5.36% decrease), and ZP [74].

### 3.4. Characterization of the Optimized Nanoemulsions after the Third Cycle of Homogenization under the Pressure of 1000 Bar

#### 3.4.1. Visual Examination and Transmittance Measurement

Depending on the droplet size of the dispersed phase, nanoemulsion may be clear when d < 50 nm or cloudy when 50 nm < d < 200 nm [10]. Visual examination revealed a cloudy, whitish appearance of homogenous formulations obtained after three cycles of HPH at 1000 bar, with no visible signs of FLZ sedimentation. According to the literature, when the PS exceeds 100 nm, nanoemulsions appear white as a result of significant multiple light scattering [67,75]. Table 2 summarizes the results of the average percentage transmittance of the prepared nanoemulsions. The transmittance values of the respective nanoemulsions after dilution with 1:10 *v*/*v* and 1:500 *v*/*v* in deionized water ranged from 27.82 to 64.40% and from 88.81 to 99.74%, respectively. A percentage of transmittance approaching 100% indicates the isotropy of the analyzed formulations [40].

#### 3.4.2. Refractive Index (RI) Measurement

The refractive index is used to characterize the isotropic nature of nanoemulsions [40], and when its value is close to the water index (RI = 1.333), nanoemulsions are considered transparent [76]. In addition, refractive index measurements of eye drops are used to detect potential visual impairment or patient discomfort after application. The RI of the tear fluid ranges from 1.340 to 1.360, so it is recommended that ophthalmic preparations have refractive index values no higher than 1.476 [52,74]. The RI of the prepared nanoemulsions were within acceptable range values (1.362–1.385, Table 2).

#### 3.4.3. Zeta Potential (ZP) Determination

The zeta potential is an important parameter that indicates the surface charge of particles and the degree of repulsion between neighboring similarly charged particles in the dispersed phase [25,77]. Higher values of the zeta potential, both (+) and (−), indicate long-term stability and protect the particles of the dispersed phase from aggregation [78]. The nanodroplets of the prepared formulations were negatively charged, and the ZP values ranged from −16.96 to −34.81 mV (Table 2). A negative ZP value is the result of the dissociation of the carboxyl group of OA in an acidic environment, which gives nanoparticles a negative charge [24,79]. Formulations with T80 and T20 showed a lower ZP than nanoemulsions with KOL, and this may likely be due to hydrogen bonding at the ether–oxygen site of the polyoxyethylene chain, with subsequent oxonium ion formation in these surfactants [74,80,81]. According to the literature, anionic nanoemulsions can be effective ophthalmic drug delivery systems, characterized by the prolonged release and increased bioavailability of active pharmaceutical ingredients [74,82,83].

#### 3.4.4. pH Measurement

The pH of well-tolerated eye drops ranges from 3.5 to 8.5, preferably characterized by a pH of 7.2 [8,44,52]. The pH values of the developed nanoemulsions were in a favorable range (3.79–4.78, Table 2). Clinical studies have shown that tested nanoemulsions with pH values in a range of 4.2–7.3 did not cause irritation to rabbit eyes [44,52], and the formulations tested ex vivo did not cause any changes on the corneal surface of the animals [8,21,84].

#### 3.4.5. Osmolality Determination

The literature data indicate that the osmolality of the lachrymal fluid is between 280 and 293 mOsm/kg upon awakening and can vary between 231 and 446 mOsm/kg as a result of the evaporation of the open eye surface [44]. Haße et al., in their evaluation of microemulsions, using the Draize test on rabbit eyes, showed no irritant effect, even with formulation osmolality in a range of 650–2400 mOsm/kg [85]. The osmolality values of the prepared formulations ranged from 20.67 to >2000 mOsm/kg for the nanoemulsions NE4_KOL_PG10, NE9_T20_PG10, and NE14_T80_PG10, containing 10% *w*/*w* propylene glycol as a surfactant (Table 2). The lowest osmotic pressure, below 100 mOsm/kg, was recorded for the NE1_KOL, NE6_T20, and NE11_T80 formulations without a cosurfactant, used only as comparative preparations. The addition of a cosurfactant, PG or PEG 200, significantly increased osmotic pressure values, i.e., nanoemulsions with PG were characterized by twofold higher values compared to the same composition with PEG 200 [85].

#### 3.4.6. Surface Tension Measurement

The results of the surface tension measurements of the nanoemulsions are summarized in Table 2. It should be noted that a low surface tension guarantees a good spreading effect of a formulation over the cornea and mixing with the components of the precorneal film, thus increasing the contact between the drug and corneal epithelium [21,52,85,86]. The surface tension of the investigated nanoemulsions ranged from 34.38 to 37.43 mN/m and was close to the physiological lachrymal fluid value of 40–50 mN/m [87].

#### 3.4.7. Viscosity Determination

Viscosity is an important parameter that determines the residence time of the drug on the ocular surface and the bioavailability of the drug from the ophthalmic product [58,88]. The viscosity of eye drops must not exceed 20.0 mPa×s [52], but for maximum penetration through the cornea, viscosity can occur in a range of 15–150 mPa×s [89]. Application products with low viscosity values provide good tolerance without pain during blinking [27,52,90]. The resulting nanoemulsions’ viscosity at a shear rate of 750 s^−1^ at 25 °C ranged from 4.02 to 26.71 mPa×s and decreased at 34 °C to 3.05–17.75 mPa×s. The results of the viscosity tests at 25 °C and 34 °C are presented in SI (Figure S3).

### 3.5. Stability Study

Nanoemulsions are characterized by much better stability against gravitational separation, flocculation, or coalescence than conventional emulsions and the relatively small size of the dispersed particles, meaning that Brownian motions are sufficient to overcome the force of gravitational separation [91,92,93]. However, nanoemulsions can destabilize during storage because they are not thermodynamically stable systems [94]. The main process that expands the particle size of the dispersed phase in nanoemulsions over time is Ostwald ripening [93]. This process arises from the difference in the solubility between droplets of different sizes, wherein larger droplets increase their size at the expense of smaller ones due to molecular diffusion through the continuous phase [95].

**Figure 4:**
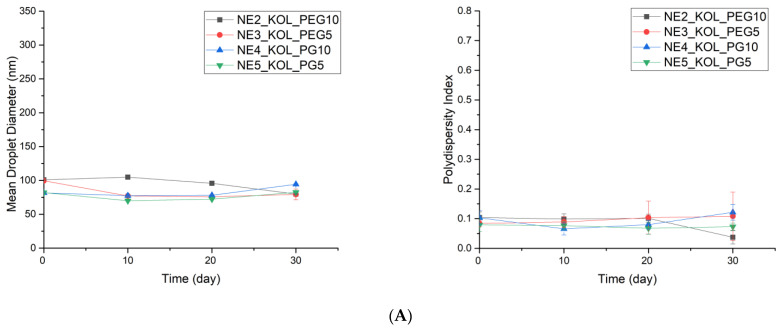

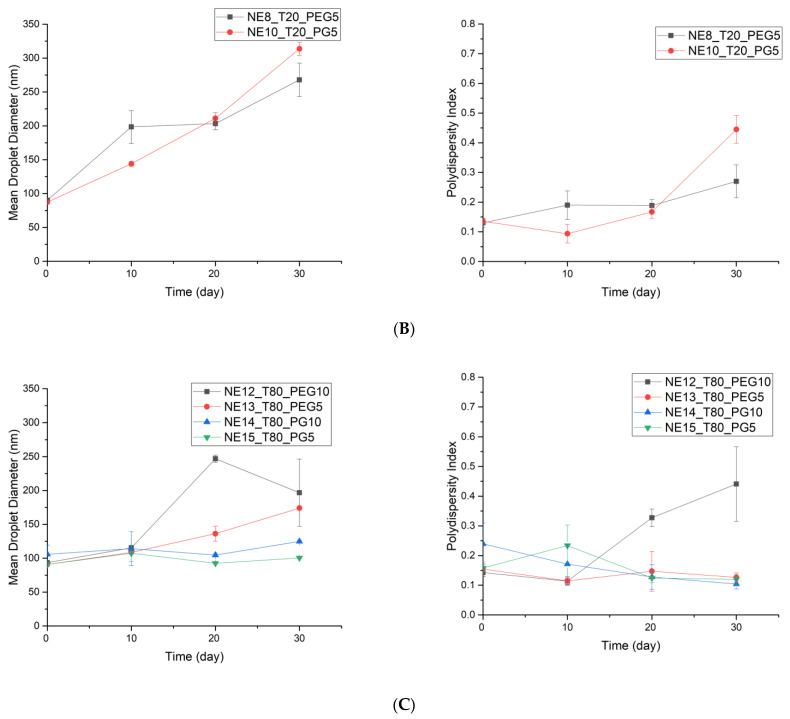
The stability data for the selected formulations over 30 days of storage at 25 °C. (**A**) With KOL; (**B**) with T20; (**C**) with T80 as surfactants.

In the conducted stability study, all nanoemulsions containing KOL as a surfactant and formulations with T80 or PG (NE14_T80_PG10 and NE15_T80_PG5) as a cosurfactant showed high stability, as confirmed via the visual assessment of the homogenous appearance, color, and the absence of precipitation after storage for 30 days at 25 °C. Favorably, no significant changes in the PS and PDI values were observed in these formulations (Figure 4), and eventually, these compositions were selected for further studies. Formulations with T80 as a surfactant, NE12_T80_PEG10 and NE13_T80_PEG5, after 30 days of storage, showed changes in appearance upon visual observation, with a significant increase in the mean PS and PDI values. At room temperature, nanoemulsions with T20 had the greatest increase in PS (44.08–259.35%) and PDI (107.69–547.88%) values, indicating high instability and phase separation in these formulations. These formulations were excluded from further studies. The stability test results of various selected nanoemulsions are presented in the SI (Table S2).

Additionally, visual analysis and evaluation of the particle size and polydispersity index were performed for the stable preparations with Kolliphor EL as a surfactant after 15 months of storage at 25 °C. In these formulations, no significant changes in the PS and PDI values were observed. These nanoemulsions maintained homogeneity without the presence of precipitation after 15 months of storage at 25 °C. Formulations with T80 as a surfactant, NE14_T80_PG10 and NE15_T80_PG5, showed signs of phase separation upon storage. The results of the 15-month stability test for the selected nanoemulsions are presented in SI (Figure S4).

Sedimentation was observed at 6 °C in the nanoemulsions containing KOL and T80. This is likely related to the pour point of OA at 16 °C [96] when the oil becomes cloudy. Moreover, all formulations showed a significant increase in the droplet size and PDI values, visual changes in transparency, or signs of phase separation after storage for 30 days at 37 °C (SI, Table S2). The destabilizing effect of Ostwald ripening can be delayed by keeping nanoemulsions at an optimal storage temperature [68]. In this study, the optimal storage temperature for the prepared nanoemulsions was established as 25 °C, as the formulations retained the smallest particle size, lowest PDI values, and satisfactory stability for 30 days at this temperature.

### 3.6. Evaluation of the Properties of the Nanoemulsions Selected in the Stability Test

#### 3.6.1. The Stability Analysis via NMR Spectroscopy

NMR spectroscopy has been used so far to determine the presence of interactions of FLZ with different components of nanoemulsions (oil, surfactant, or cosurfactant) [84,97]. In this work, the NMR technique was utilized to follow the occurrence of alterations in nanoemulsions upon storage, at the molecular level. The changes regarding the lineshape, the integral values (INTs), and the full width at half maximum (FWHM) of the NMR resonance peaks corresponding to either the oil/surfactant phase or drug molecules were evaluated as indicators of the nanoemulsions’ stability. The widening and lowering intensity of the peaks might indicate the aggregation process between the respective components. The lower ratio of absolute integral values and the higher ratio of FWHM between samples after the stability test (series 2) and freshly prepared samples (series 1) characterized the compositions with lower stability. The 1D NMR spectra of the fluconazole-loaded nanoemulsions (series 1) compared to the blank series of the corresponding formulations did not differ from each other in the spectrum area representing OA, surfactants, and cosurfactants (S_mix_), signalizing that the addition of the drug did not affect the remaining components of the nanoemulsions.

**Figure 5:**
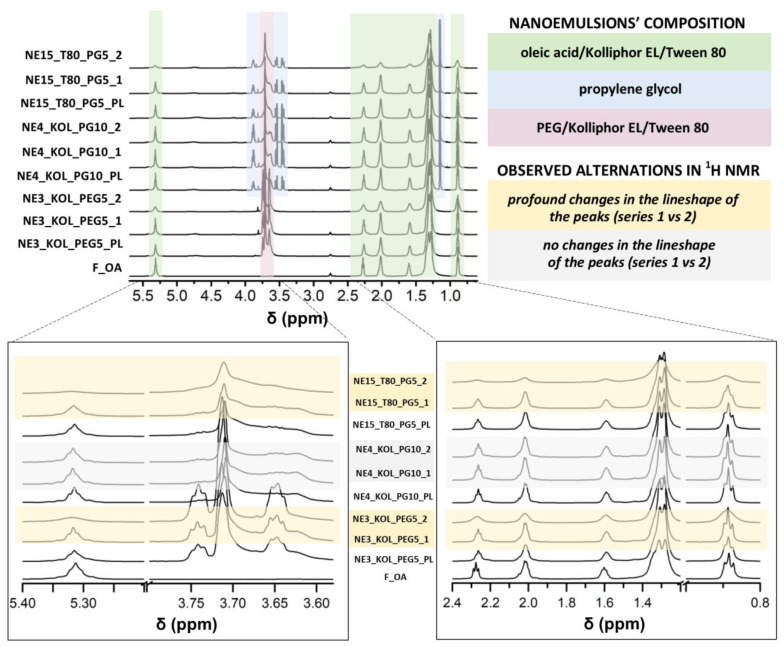
^1^H NMR spectra (insert with acetone-d_6_, 298 K, 600.13 MHz for ^1^H) of NE3_KOL_PEG5, NE4_KOL_PG10, and NE15_T80_PG5 (blank series, series 1, and series 2), and F_OA (oleic acid and S_mix_ region).

When looking at the lineshape of the peaks assigned to OA, KOL, and T80 in the 1D NMR spectra of both series of formulations with FLZ, the strongest changes were observed for NE3_KOL_PEG5, NE14_T80_PG10, and NE15_T80_PG5 (Figure 5). This confirms that T80 chosen as a surfactant and a low concentration of PEG 200 as a cosurfactant were less stabilizing components for the investigated nanoemulsions. The peaks of OA and KOL in NE3_KOL_PEG5_2 were widened in contrast to NE2_KOL_PEG10_2 (the ratio of FWHM_series2/series1_ in the ranges of 1.46–2.34 and 0.99–1.01, respectively), meaning that a lower concentration of PEG 200 was less efficient in retaining the stability of the formulation. On the contrary, the lineshape of the oil phase and surfactant mixture phase in NE4_KOL_PG10 and NE5_KOL_PG5 (series 1 vs. series 2) remained mostly unchanged (Figure 5), which might be explained by a better stabilizing effect of PG in comparison to PEG 200. In the formulations containing T80, the alterations in the shape and the decreasing intensity (in the following order: blank series > series 1 > series 2) of the OA and T80 peaks included both NE14_T80_PG10 and NE15_T80_PG5. This was observed to a larger extent in the latter (the ratio of INT_series2/series1_ in the ranges of 0.91–1.00 and 0.45–0.88, respectively), proving that, in general, the addition of T80 resulted in less stable nanoemulsions, whereas a higher concentration of PG, in this sample, stabilized the formulation in a more effective way. Some studies have shown that the larger the difference in the size of the head groups of the applied surfactants and cosurfactants, the greater the synergistic effects on stabilizing emulsions. Low-molecular-weight cosurfactants are able to pack more efficiently along with high-molecular-weight surfactant molecules at the oil–water interface [19,98].

**Figure 6:**
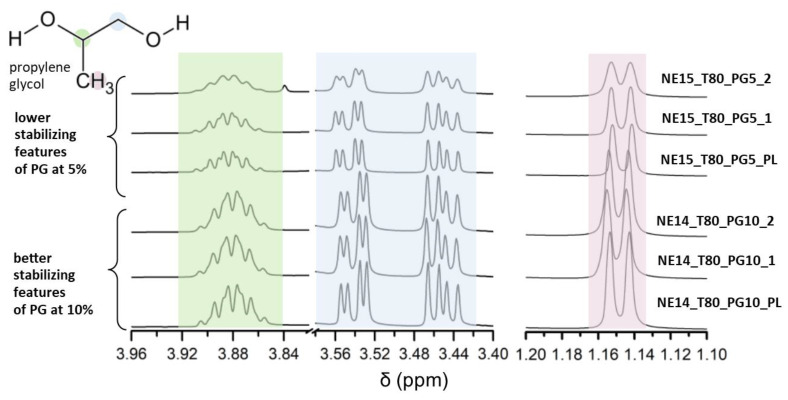
^1^H NMR spectra (insert with acetone-d_6_, 298 K, 600.13 MHz for ^1^H) of NE14_T80_PG10 and NE15_T80_PG5 (blank series, series 1, and series 2; propylene glycol region).

Overall, the intensity of the OA and S_mix_ peaks remained the same or were reduced throughout the NMR spectra of the formulations when series 2 and series 1 were compared. The only absorption signal that was largely increased was at 3.7 ppm in the case of NE2_KOL_PEG10, NE3_KOL_PEG5, and NE15_T80_PG5, corresponding to the structure part of PEG 200, KOL, and T80. The increasing integral in the samples after the stability test, in comparison to the freshly prepared samples (ratios of 1.43, 1.25, and 1.08), might be a result of the increased proportion of free polyethylene groups appearing during the storage of the nanoemulsion. The formulations NE3_KOL_PEG5 and NE15_T80_PG5 were characterized by the lowest pH values among the investigated compositions (3.86 ± 0.08 and 3.94 ± 0.14, respectively; Table 2). Taking into account the pH values, it is suspected that the hydrolysis of the ester bond in KOL and T80 was broken, resulting in the appearance of polyethylene groups. Considering all formulations containing PG as a cosurfactant, the lineshape and the intensity of the corresponding peaks (namely, at 1.3 ppm, 3.4–3.5 ppm, and 3.8 ppm) in nanoemulsions with the addition of 10% PG (NE4_KOL_PG10 and NE14_T80_PG10) were less perturbed (compared to, e.g., NE15_T80_PG5), confirming, as described above, that a higher concentration of PG exhibited better stabilizing features (Figure 6).

**Figure 7:**
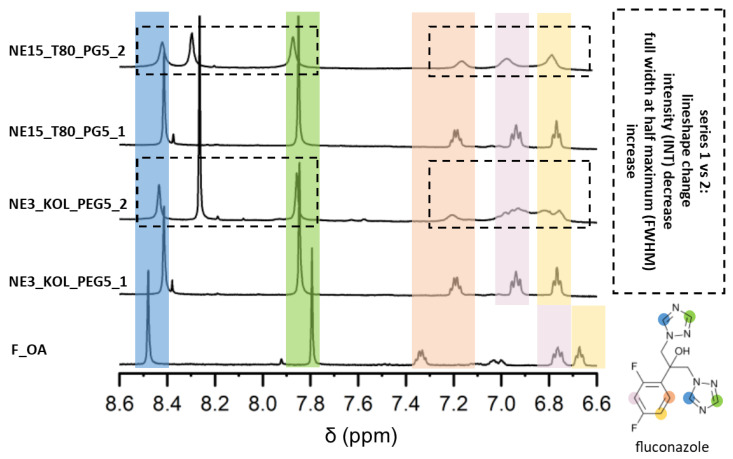
^1^H NMR spectra (insert with acetone-d_6_, 298 K, 600.13 MHz for ^1^H) of NE3_KOL_PEG5, NE15_T80_PG5 (series 1 and series 2), and F_OA (fluconazole region).

In order to investigate the stability of the drug in the formulations, the FWHM and INT values were calculated for FLZ peaks in series 1 and 2. Significant increases in the FWHM_series2_/_series1_ ratio were observed in T80 nanoemulsions and NE3_KOL_PEG5 (1.52–1.64), then in the NE2_KOL_PEG10 (1.00–3.51) and NE5_KOL_PG5 (0.98–2.74) formulations, ending with the smallest values characterizing the NE4_KOL_PG10 nanoemulsion (1.04–2.13). The smallest values for the INT_series 2/series 1_ ratio were observed in the NE3_KOL_PEG5 (0.16–0.63) and NE15_T80_PG5 (0.31–1.07) formulations, exhibiting the largest loss in the integral values after the stability test. Overall, the drug was most stable in Kolliphor EL-loaded nanoemulsions, with the best results when it was accompanied by 10% PG, followed by the addition of 5% PG or 10% PEG 200. Similarly, the lineshape of FLZ peaks significantly changed in the case of Tween 80 nanoemulsions and NE3_KOL_PEG5 (Figure 7).

Similar findings concerning the higher stability of the formulations loaded with KOL over T80 as a surfactant and PG over PEG 200 as a cosurfactant were observed when the NMR spectra of the samples after the stability test (series 2) were taken into consideration. Namely, the intensity of the peaks assigned to FLZ was the same when NE4_KOL_PG10_2 and NE5_KOL_PG5_2 were compared, partially unchanged when NE2_KOL_PEG10_2 and NE3_KOL_PEG5_2 were collated, and significantly reduced in the case of a comparison between NE14_T80_PG10_2 and NE15_T80_PG5_2. 

Similar dependences between the composition and the stability of the formulations were observed in the 2D NMR results (SI, Figure S5). In the case of the nanoemulsions with PEG 200 as a cosurfactant (NE2_KOL_PEG10 and NE3_KOL_PEG5), after the stability test (series 2), there were fewer cross-peaks between the drug and other components, and those present were characterized by a lower intensity in comparison to the samples measured directly after preparation (series 1). Especially in the NE3_KOL_PEG5 nanoemulsion, the effect was visible, meaning that the addition of 10% PEG 200 ensures the stability of the formulation to a greater extent than 5% PEG 200 (SI, Figure S6a). On the contrary, an analogical set of cross-peaks remained unchanged when series 1 and series 2 compared in nanoemulsions stabilized with PG (NE4_KOL_PG10 and NE5_KOL_PG5; SI, Figure S6b). This confirms that KOL accompanied with PG resulted in more stable emulsions than when PEG 200 was added. Furthermore, when comparing the NE14_T80_PG10 and NE15_T80_PG5 samples, the strongest changes in the cross-peak set after the stability test were seen in the case of the formulation with 5% PG added (SI, Figure S6c). Therefore, comparing changes in NOESY spectra in the course of studying the stability of nanoemulsions with the same cosurfactant (PG) and different surfactants (KOL vs. T80), it might be concluded that KOL ensured higher stability in comparison to T80. Interestingly, when looking at the cross-peaks between the signals of OA and S_mix_, they exhibited a smaller intensity in the samples after the stability test compared to the samples measured just after preparation in NE2_KOL_PEG10 and NE3_KOL_PEG5. The effect was especially visible in the latter one, confirming that emulsions with PEG 200 as a cosurfactant were the least stable among the investigated compositions.

NMR spectroscopy can also be applied to determine the interactions between drug molecules and nanoemulsion ingredients, namely, the location or partitioning of the drug between the oil and water phases [97,99]. In our study, in NOESY spectra, all cross-peaks between the drug and both the oil and surfactant phases exhibited the same sign as the diagonal, viz. negative NOE. Such observations for the small molecule in the presence of large molecules, including the oil phase and surfactants’ mixture, indicate the close proximity of drug molecules to the abovementioned phases. As there was an overlap in the Kolliphor EL, Tween 80, and oleic acid peaks, the indication of exactly where the interactions take place remained ambiguous. FLZ protons (excluding methylene protons due to the overlap with the residual water peak) experienced cross-relaxation with methylene protons of oleic acid, Kolliphor EL, and Tween 80 (at 1.2–1.3 ppm), as well as polyethylene groups comprising a part of PEG, Kolliphor EL, and Tween 80 (3.6–3.7 ppm) in all investigated nanoemulsions. Other intermolecular contacts between the drug and other components of the nanoemulsions were less intense and mainly concerned the triazole ring in FLZ.

#### 3.6.2. Drug Content

The percentage of the drug content in the selected nanoemulsions containing FLZ ranged from 90.08 ± 1.32% to 94.30 ± 1.92%, meeting pharmacopeial acceptable limits of 90–110% [100].

#### 3.6.3. In Vitro Release Studies

**Figure 8:**
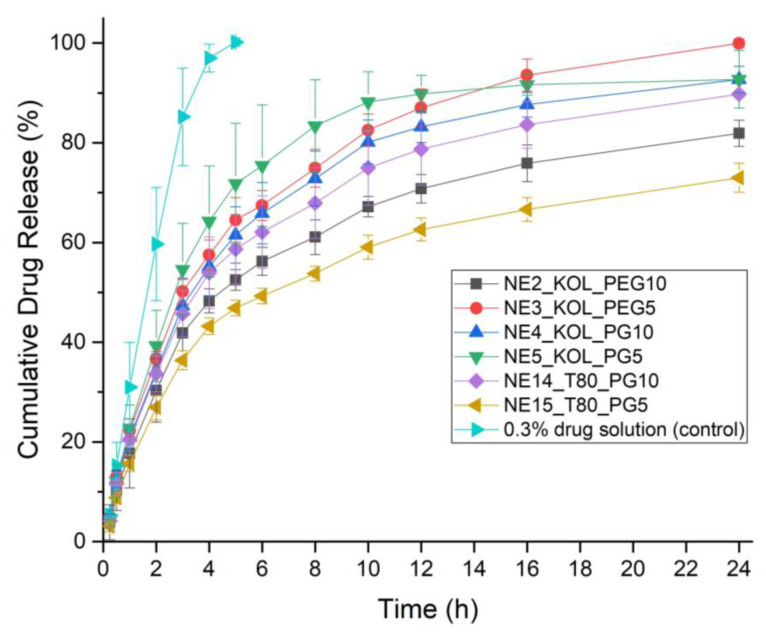
Cumulative drug release (%) of FLZ from the selected fluconazole-loaded nanoemulsions and the 0.3% aqueous drug solution being the control at 37 ± 0.5 °C in phosphate buffer (pH 7.4).

The stable formulations after the 30-day stability test at 25 °C were selected for the drug release studies. The results of FLZ release from nanoemulsions containing KOL or T80, along with different cosurfactants, PEG 200 and PG, are presented in Figure 8. A comparison of the release properties of the nanoemulsions containing the same concentrations of oil, KOL as a surfactant, and differing cosurfactants (PEG 200 and PG) revealed that the release efficiency of these nanoemulsions at the end of a 24-h period followed the order NE3_KOL_PEG5 > NE5_KOL_PG5 > NE4_KOL_PG10 >  NE2_KOL_PEG10. FLZ release from the nanoemulsions containing KOL and 5% of the cosurfactants (NE3_KOL_PEG5 and NE5_KOL_PG5) was higher than from the nanoemulsions containing 10% of the cosurfactants (NE4_KOL_PG10 and NE2_KOL_PEG10). This may be due to the lower viscosity of the formulations containing 5% of cosurfactants (5.53 mPa×s in the case of NE3_KOL_PEG5 and 6.67 mPa×s in the case of NE5_KOL_PG5) compared to a 10% cosurfactant content (10.62 mPa×s for NE4_KOL_PG10 and 12.62 mPa×s for NE2_KOL_PEG10). However, comparing the release of FLZ from the nanoemulsion with T80 containing 5% and 10% *w*/*w* PG, a significant difference was observed between their release efficiencies, namely, 72.99% for NE15_T80_PG5 (viscosity 12.84 mPa×s) and 89.75% for NE14_T80_PG10 (viscosity 12.07 mPa×s), at the end of a 24-h period, despite the similar viscosity values. The obtained release curves (Figure 8) showed that the optimized FLZ nanoemulsions exhibit a satisfactory sustained drug release behavior compared to the total drug release from the reference solution at 5 h. The difference in the release pattern between the nanoemulsions and the solution indicates that these nanoemulsions can act as a drug reservoir, as their viscosity values were 5.53–12.84 mPa×s compared to the solution’s 0.96 mPa×s.

#### 3.6.4. Antifungal Activity Test

For the antifungal activity testing, the nanoemulsions containing KOL as a surfactant were selected characterized by the longest stability over 2 months of storage at 25 °C. The inhibition zones of *C. albicans*, *C. parapsilosis*, *C. glabrata*, and *C. tropicalis* produced by the selected nanoemulsions were in the ranges of 34 ± 4 to 36 ± 5 mm, 30 ± 2 to 34 ± 1 mm, 14 ± 2 to 17 ± 1 mm, and 34 ± 5 to 37 ± 2.5 mm, respectively. In the case of the drug solution, the inhibition zones of *C. albicans*, *C. parapsilosis*, *C. glabrata*, and *C. tropicalis* were 35 ± 4 mm, 30 ± 5 mm, 17 ± 2.5 mm, and 37 ± 2.5 mm, respectively. The obtained inhibition zones were not significantly different from those measured for the aqueous FLZ solution (*p* >0.5) at the same concentration as present in nanoemulsions. The diameters of the fungal growth inhibition zones are shown in SI (Table S3).

Considering that the examined formulations had higher viscosity and prolonged release compared to the aqueous solution of the drug, it might be concluded that the release of the drug was delayed in the nanoemulsions compared to the control sample under the conditions of the microbiological assay. Moreover, in vitro antifungal activity showed that neither the HPH process nor the components of the formulations affected the antifungal efficacy of neat FLZ.

#### 3.6.5. Surface Morphology—Transmission Electron Microscopy (TEM)

**Figure 9:**
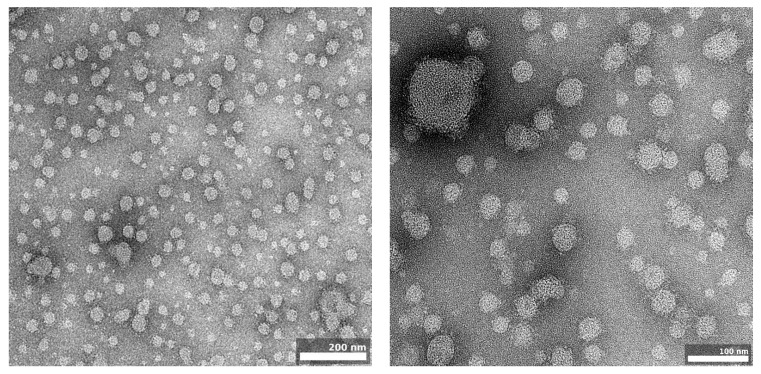
Representative negative stained (2% uranyl acetate) TEM images of the fluconazole-loaded nanoemulsion NE5_KOL_PG5.

The use of transmission electron microscopy enabled the observation of the microstructures of the selected nanoemulsions and their morphology [77]. Figure 9 shows the spherical droplets of the NE5_KOL_PG5 nanoemulsion, which appeared bright against a dark background. The measured size of the dispersed phase droplets in the images was consistent with the results obtained from the particle size measurements via DLS and was less than 200 nm.

This study has a few limitations to note. Our study is an analysis of the technological process and effect of various homogenization parameters on the effective preparation of stable nanoemulsions for potential application on the eyeball with antifungal drugs. The researchers have not carried out in vivo studies demonstrating ocular tolerability and bioavailability due to the cost factor and ethical considerations. However, pharmaceutical ingredients classified as practically non-irritant and applied in a wide range of concentrations in ophthalmic formulations were used in our tests. Additionally, we performed in vitro tests, providing a more controlled and standardized environment for testing than an in vivo study, which can reduce the variability in the results.

## 4. Conclusions

Optimized ophthalmic nanoemulsions should have the following characteristics: (1) an average droplet diameter <200 nm, (2) high stability during storage, (3) a low S_mix_ concentration, and (4) a sufficient amount of oil for the effective drug-loaded formulation. HPH is a promising method for the preparation of stable ophthalmic formulations with suitable physicochemical properties, but its use still needs to be improved by the further optimization of process variables. In the current study, we successfully investigated the feasibility of obtaining fluconazole-loaded nanoemulsions via HPH containing 20% *w*/*w* of OA as an oil phase in combination with only 10% *w*/*w* of surfactants (KOL, T80, and T20) and 5–10% *w*/*w* of cosurfactants (PG and PEG 200). This study showed that the applied pressure, the number of cycles, and the homogenization outlet temperature had a significant effect on the average PS, size distribution, and PDI values of the investigated nanoemulsions. After the optimization of homogenization parameters, three cycles at an operating pressure of 1000 bar were selected as favorable process conditions. Sterilizable fluconazole-loaded nanoemulsions were obtained with an unimodal particle size distribution, an average droplet diameter in the range of 80.63–129.68 nm, PDI values below 0.25, and an optimum outlet temperature of the formulations of 35.0–36.6 °C. In all cases, increasing the concentrations of PG and PEG 200 was expected to reduce the droplet size, but in the case of the formulations with T20 as a surfactant, with the addition of 10% *w*/*w* of cosurfactants during homogenization, “over-processing” was observed, identified by the increased PS and PDI values. Furthermore, the results of the stability studies showed that the type of surfactant had a significant effect on the stability of the o/w nanoemulsions. It was observed that a higher HLB value of the used surfactants reduced the stability of the formulations in the following order KOL > T80 > T20 (HLB values of 13.5, 15, and 16.7, respectively). The NMR technique enabled an in-depth observation of the alterations occurring upon storage of the selected formulations. NMR spectra confirmed that the KOL-based formulations (~2500 g/mol) provided higher stability in the nanoemulsion composition compared to the use of T80 (~1310 g/mol) and a better stabilizing effect of PG (76.09 g/mol) as a cosurfactant was observed when compared to PEG 200 (190–210 g/mol). The stability results for the developed nanoemulsions indicate the possible use of these formulations as promising fluconazole eye delivery systems with the potential for the prolonged release of the substance with antifungal activity similar to a 0.3% fluconazole solution. Therefore, an extensive understanding of the changes that the components of the formulation undergo at the molecular level, the critical process parameters of the HPH technology, and the evaluation of the properties of the resulting compositions will allow a subsequent design of effective and stable formulations loaded with drug molecules from the azole group.

## Figures and Tables

**Table 1:** Compositions of 0.3% *w*/*w* fluconazole-loaded nanoemulsion formulations.

Formulation Code	Compositions (% *w*/*w*)										
	Oleic Acid	Surfactants (S)				Cosurfactants (Cos)		Water	% S_mix_	S:Cos Ratio	HLB/ HLB_mix_* Value
		KOL	T20	T80	PLU	PEG 200	PG				
NE1_KOL	20	10						69.7			13.50
NE2_KOL_PEG10	20	10				10		59.7	20	1:1	15.80
NE3_KOL_PEG5	20	10				5		64.7	15	2:1	15.03
NE4_KOL_PG10	20	10					10	59.7	20	1:1	11.44
NE5_KOL_PG5	20	10					5	64.7	15	2:1	12.13
NE6_T20	20		10					69.7			16.70
NE7_T20_PEG10	20		10			10		59.7	20	1:1	17.40
NE8_T20_PEG5	20		10			5		64.7	15	2:1	17.17
NE9_T20_PG10	20		10				10	59.7	20	1:1	13.04
NE10_T20_PG5	20		10				5	64.7	15	2:1	14.26
NE11_T80	20			10				69.7			15.00
NE12_T80_PEG10	20			10		10		59.7	20	1:1	16.55
NE13_T80_PEG5	20			10		5		64.7	15	2:1	16.03
NE14_T80_PG10	20			10			10	59.7	20	1:1	12.19
NE15_T80_PG5	20			10			5	64.7	15	2:1	13.13
NE16_PLU	20				10			69.7			22.00
NE17_PLU_PEG10	20				10	10		59.7	20	1:1	20.05
NE18_PLU_PG10	20				10		10	59.7	20	2:1	15.69

KOL—Kolliphor EL; T20—Tween 20; T80—Tween 80; PLU—Pluronic F127; PEG—PEG 200; PG—propylene glycol; HLB_mix_* = F_S_HLB_S_ + F_Cos_HLB_Cos_; F_s_, F_Cos_—the weight fractions.

**Table 2:** Physicochemical properties of 0.3% *w*/*w* fluconazole nanoemulsions (n = 3).

Formulation Code	Mean Droplet Diameter (nm) *		Polydispersity Index		Zeta Potential (mV)		Osmolality (mOsm/kg)		pH		Surface Tension (mN/m)		Refractive Index		T_10_ (%)		T_500_ (%)	
	*M*	*SD*	*M*	*SD*	*M*	*SD*	*M*	*SD*	*M*	*SD*	*M*	*SD*	*M*	*SD*	*M*	*SD*	*M*	*SD*
NE1_KOL	91.85	2.20	0.149	0.031	−20.39	7.72	20.67	0.58	4.42	0.05	36.03	0.34	1.3623	0.0002	64.40	0.03	99.74	0.07
NE2_KOL_PEG10	100.78	0.85	0.104	0.019	−16.96	0.70	1026.33	6.81	4.78	0.05	35.45	0.45	1.3848	0.0006	48.87	0.14	92.18	0.11
NE3_KOL_PEG5	99.39	1.07	0.084	0.011	−17.76	0.91	426.33	8.62	3.86	0.08	37.43	0.30	1.3731	0.0002	52.33	0.04	97.10	0.27
NE4_KOL_PG10	81.65	0.85	0.103	0.024	−25.48	5.73	>2000		4.62	0.22	35.03	0.46	1.3852	0.0006	53.46	0.16	98.66	0.13
NE5_KOL_PG5	82.07	0.88	0.079	0.021	−18.47	0.74	1035.33	23.35	4.33	0.05	37.25	0.45	1.3757	0.0003	54.98	0.31	98.03	0.47
NE6_T20	80.63	2.85	0.161	0.010	−27.21	3.01	70.00	1.73	4.24	0.34	36.10	0.61	1.3662	0.0002	49.18	0.60	97.28	0.21
NE7_T20_PEG10	129.68	0.99	0.094	0.019	−32.36	3.80	1238.67	24.01	4.42	0.06	36.90	0.53	1.3854	0.0001	28.87	0.10	88.81	0.26
NE8_T20_PEG5	90.25	0.91	0.130	0.015	−23.21	0.52	546.00	1.73	3.79	0.11	35.50	0.42	1.3788	0.0001	31.96	0.93	94.23	0.35
NE9_T20_PG10	98.69	0.32	0.106	0.012	−26.14	0.76	>2000		4.27	0.20	36.68	1.00	1.3812	0.0003	29.89	0.06	92.90	0.15
NE10_T20_PG5	87.32	0.64	0.136	0.010	−34.81	2.87	893.33	4.93	4.04	0.05	34.38	0.92	1.3798	0.0001	53.60	0.70	97.21	0.06
NE11_T80	95.67	1.13	0.132	0.013	−27.90	4.42	31.67	1.53	3.86	0.06	35.80	0.94	1.3662	0.0002	35.69	0.16	97.64	0.20
NE12_T80_PEG10	93.55	1.43	0.143	0.014	−25.54	1.75	1029.67	33.65	4.40	0.11	35.13	0.39	1.3828	0.0003	27.82	0.03	94.83	0.34
NE13_T80_PEG5	90.72	1.85	0.155	0.024	−28.01	1.54	446.67	15.17	4.15	0.04	36.08	0.90	1.3795	0.0004	33.03	0.10	93.59	0.30
NE14_T80_PG10	105.89	13.32	0.240	0.070	−29.73	1.82	>2000		4.22	0.12	35.93	0.47	1.3841	0.0003	31.08	0.05	92.55	0.42
NE15_T80_PG5	90.73	0.93	0.158	0.014	−28.11	0.65	874.67	16.74	3.94	0.14	35.55	0.39	1.3714	0.0004	32.99	0.15	98.69	0.32

* The mean droplet diameter for the nanoemulsion after the third cycle of homogenization under a pressure of 1000 bar. T_10_ (%)—transmittance after dilution in deionized water 1:10 *v*/*v*; T_500_ (%)—transmittance after dilution in deionized water 1:500 *v*/*v*; M—mean; SD—standard deviation.

## Data Availability

Data are contained within the article and Supplementary Materials.

## Supplementary Materials

The following supporting information can be downloaded at: https://www.mdpi.com/article/10.3390/pharmaceutics16010011/s1, Figure S1. The droplet size distribution by intensity of NE15_T80_PG5 formulation after 10 passes through the homogenizer at an operating pressure of 1000 bar obtained via the dynamic light scattering method; Figure S2. The effect of the sterilization method—filtration—on the particle size of the prepared nanoemulsions. (A) With Kolliphor; (B) with Tween 20; (C) with Tween 80 as surfactants; Figure S3. Comparison of the viscosity of the selected nanoemulsions at 25 °C and 34 °C. (A) With Kolliphor; (B) with Tween 20; (C) with Tween 80 as surfactants; Figure S4. The stability data for the selected formulations with Kolliphor EL as the surfactant after 15 months of storage at 25 °C. (A) Mean droplet diameter; (B) polydispersity index values; Figure S5. ^1^H NMR spectra of NE4_KOL_PG10, NE5_KOL_PG5, NE2_KOL_PEG10, NE3_KOL_PEG5, NE14_T80_PG10, and NE15_T80_PG5 (series 2, fluconazole region); Figure S6. ^1^H-^1^H NOESY NMR spectra of NE3_KOL_PEG5, NE4_KOL_PG10, NE15_T80_PG5 (series 1 presented in black and series 2 presented in blue); Table S1. The solubility of fluconazole in the selected oils, surfactants, cosurfactants, and water (mean ± SD, n = 3); Table S2. The stability testing results of different selected nanoemulsion formulations; Table S3. The antifungal activity of the 0.3% *w*/*w* fluconazole solution, optimal fluconazole-loaded nanoemulsions, and blank formulations determined via the disc diffusion method.
