# Supplementary material for: The Effect of High-Pressure Homogenization Conditions on the Physicochemical Properties and Stability of Designed Fluconazole-Loaded Ocular Nanoemulsions
Source: Pharmaceutics. 2023 Dec 20;16(1):11. doi: 10.3390/pharmaceutics16010011 (PMC10818809; doi:10.3390/pharmaceutics16010011)
Supplement: Supplementary file 1 [file pharmaceutics-16-00011-s001.zip › pharmaceutics-2624406-supplementary.pdf]

Supplementary Information

The Effect of High-Pressure Homogenization Conditions on the Physicochemical Properties and Stability of Designed Fluconazole-Loaded Ocular Nanoemulsions

Agnieszka Gawin-Mikołajewicz [1,]*, Urszula Nawrot [2], Katarzyna Hanna Malec [1], Karolina Krajewska [1], Karol Przemysław Nartowski [1,†] and Bożena Lucyna Karolewicz [1]

[1] Department of Drug Form Technology, Wroclaw Medical University, 211A Borowska Str.,
50-556 Wroclaw, Poland; k.malec@umw.edu.pl (K.H.M.); karolina.krajewska@student.umw.edu.pl (K.K.); karol.nartowski@umw.edu.pl (K.P.N.); bozena.karolewicz@umw.edu.pl (B.L.K.)

[2] Department of Pharmaceutical Microbiology and Parasitology, Wroclaw Medical University,
211A Borowska Str., 50-556 Wroclaw, Poland; urszula.nawrot@umw.edu.pl

* Correspondence: agnieszka.gawin-mikolajewicz@umw.edu.pl; Tel.: +48-71-78-40-314

† deceased author.

Table S1. The solubility of fluconazole in the selected oils, surfactants, cosurfactants and water (mean ± SD, n=3).

Type of component	Excipient	Solubility (mg/mL)	
**Oil**	Oleic acid	13.42 ± 0.007	
	Isopropyl myristate	0.68 ± 0.092	
	Cottonseed oil	0.41 ± 0.005	
	Sesame oil	0.38 ± 0.024	
	Castor oil	0.37 ± 0.001	
	Peanut oil	0.22 ± 0.005	
	Olive oil	0.20 ± 0.013	
**Surfactant**			**HLB value**
	Pluronic F127 (10% solution in water)	9.95 ± 0.008	22.0
	Tween 20	5.10 ± 0.026	16.7
	Tween 80	2.50 ± 0.002	15.0
	Kolliphor EL	1.64 ± 0.006	13.5
**Cosurfactant**	Propylene glycol	25.60 ± 0.034	9.37, 9.38
	PEG 200	24.77 ± 0.015	18.1
	Span 80	1.43 ± 0.084	4.3
**Water**		4.90 ± 0.010	

Table S2. The stability testing result of different selected nanoemulsion formulations.

Formulation Code	10 days			20 days			30 days			Inference
	**6°C**	**25°C**	**37°C**	**6°C**	**25°C**	**37°C**	**6°C**	**25°C**	**37°C**	
**NE2_KOL_PEG10**	S	-	-	S	-	W	S	-	W	**Passed**
**NE3_KOL_PEG5**	S	-	W	S	-	W	S	-	W	**Passed**
**NE4_KOL_PG10**	S	-	W	S	-	W	S	-	W	**Passed**
**NE5_KOL_PG5**	S	-	W	S	-	W	S	-	W	**Passed**
**NE7_T20_PEG10**	-	-	-	-	D	D	-	PS	PS	Failed
**NE8_T20_PEG5**	W	W	D	W	W	PS	W	W	PS	Failed
**NE9_T20_PG10**	-	-	D	W	W	PS	-	PS	PS	Failed
**NE10_T20_PG5**	-	-	W	-	W	D	-	W	PS	Failed
**NE12_T80_PEG10**	-	-	-	S	W	D	S	W	D	Failed
**NE13_T80_PEG5**	S	-	D	S	-	PS	S	W	PS	Failed
**NE14_T80_PG10**	-	-	W	-	-	D	W	W	PS	**Passed**
**NE15_T80_PG5**	W	-	W	-	-	W	W	-	W	**Passed**

– no changes, W—white, S—sediment, PS—phase separation, D—delamination

Table S3. An antifungal activity of the 0.3% *w/w* fluconazole solution, optimal fluconazole-loaded nanoemulsions and blank formulations determined by the disc diffusion method.

Formulation Code	Diameter of inhibition zone (mm)			
	C. albicans ATCC 90028	*C. parapsilosis* ATCC 90018	*C. glabrata* ATCC 90030	*C. tropicalis* ATCC 750
0.3% fluconazole solution	35 ± 4	30 ± 5	17 ± 2.5	37 ± 2.5
NE2_KOL_PEG10	34 ± 4	30 ± 2	16 ± 1.5	37 ± 2.5
NE2_KOL_PEG10 (BF)	6*	6*	6*	6*
NE3_KOL_PEG5	34 ± 4	34 ± 1	16 ± 2	34 ± 5
NE3_KOL_PEG5 (BF)	6*	6*	6*	6*
NE4KOLPG10	36 ± 5	32 ± 2	17 ± 1	34 ± 5
NE4_KOL_PG10 (BF)	6*	6*	6*	6*
NE5_KOL_PG5	35 ± 4	32 ± 2	14 ± 2	34 ± 5
NE5_KOL_PG5 (BF)	6*	6*	6*	6*

BF—Blank Formulations; *—paper discs diameter

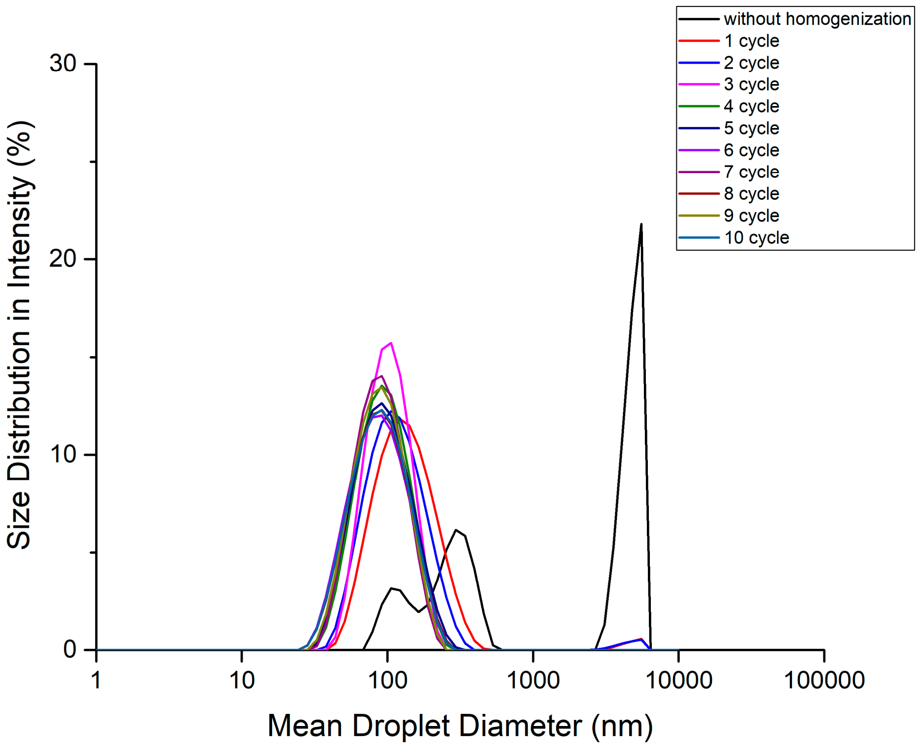

Figure S1. The droplet size distribution by intensity of NE15_T80_PG5 formulation after 10 passes through the homogenizer at the operation pressure of 1000 bar obtained by the dynamic light scattering method.

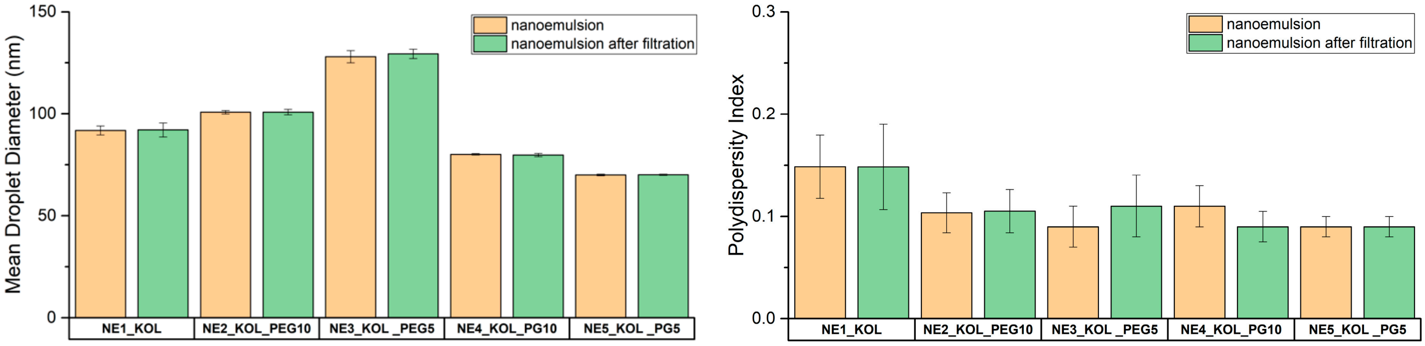

(**A**)

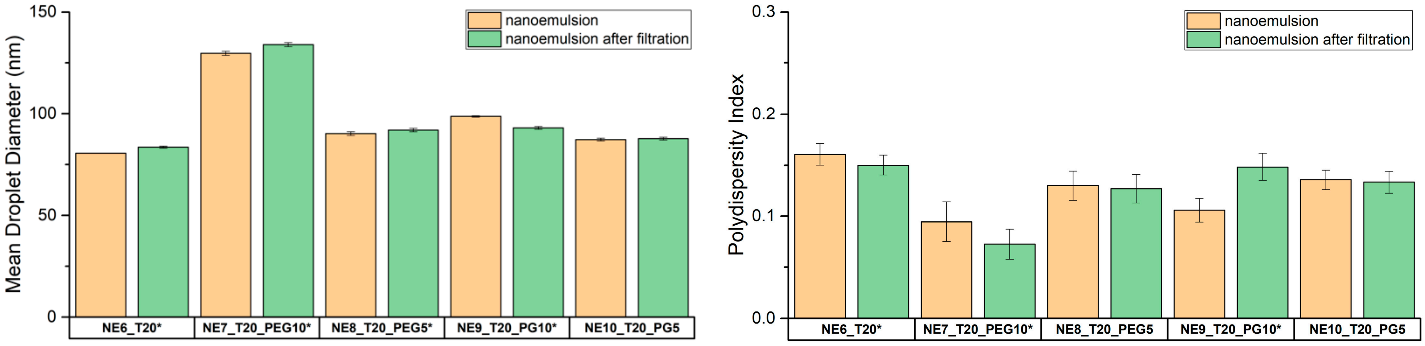

(**B**)

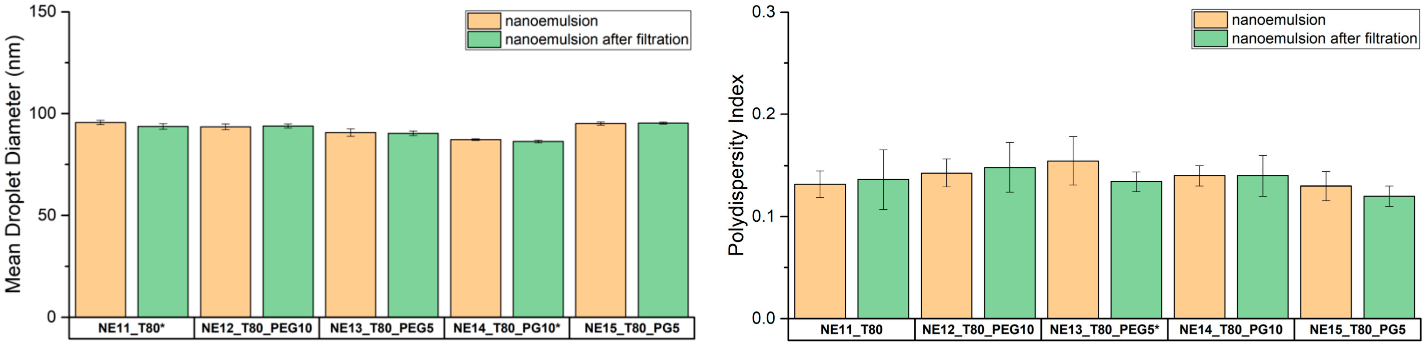

(**C**)

*the nanoemulsions which values of $p<0.05$ were considered statistically significant

Figure S2. The effect of sterilization method—filtration, on the particle size of the prepared nanoemulsions: (**A**) with Kolliphor; (**B**) with Tween 20; (**C**) with Tween 80 as surfactants.

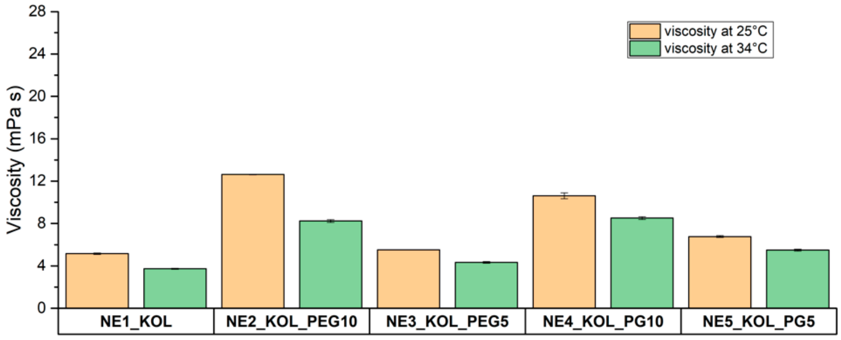

(**A**)

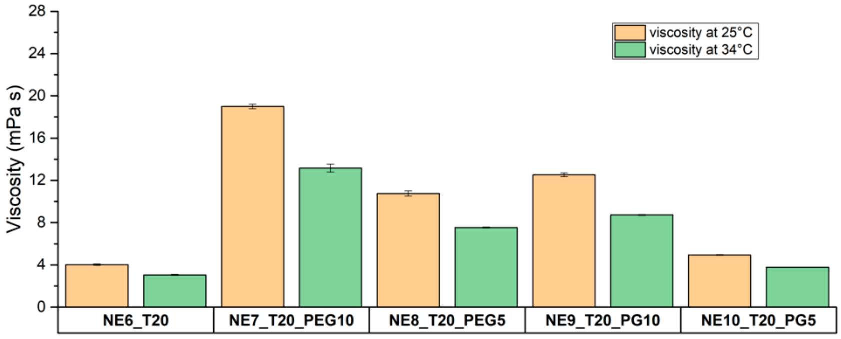

(**B**)

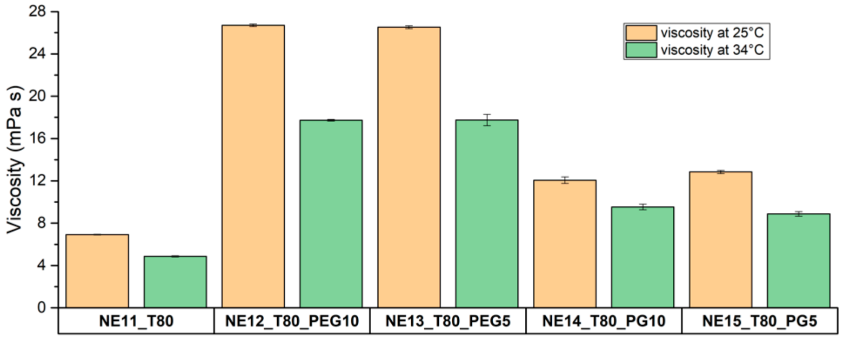

(**C**)

Figure S3. Comparison of the viscosity of the selected nanoemulsions at 25 °C and 34 °C: (**A**) with Kolliphor; (**B**) with Tween 20; (**C**) with Tween 80 as surfactants.

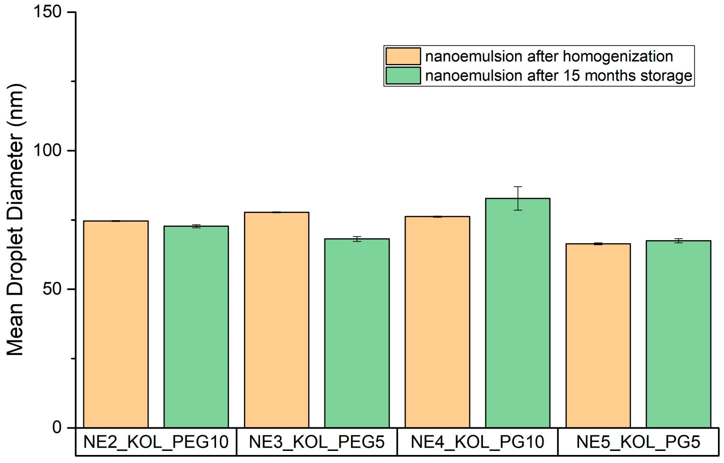

(**A**)

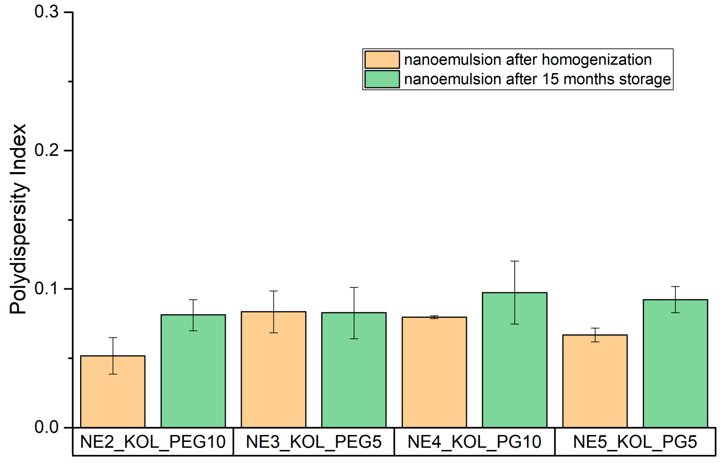

(**B**)

Figure S4. The stability data for the selected formulations with Kolliphor EL as the surfactant after the 15 months storage at 25 °C: (**A**) mean droplet diameter; (**B**) polydispersity index values.

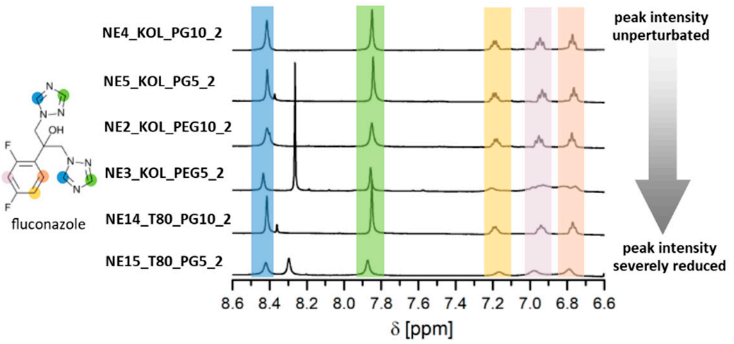

Figure S5. ^{1}H NMR spectra of NE4_KOL_PG10, NE5_KOL_PG5, NE2_KOL_PEG10, NE3_KOL_PEG5, NE14_T80_PG10, and NE15_T80_PG5 (series 2, fluconazole region).

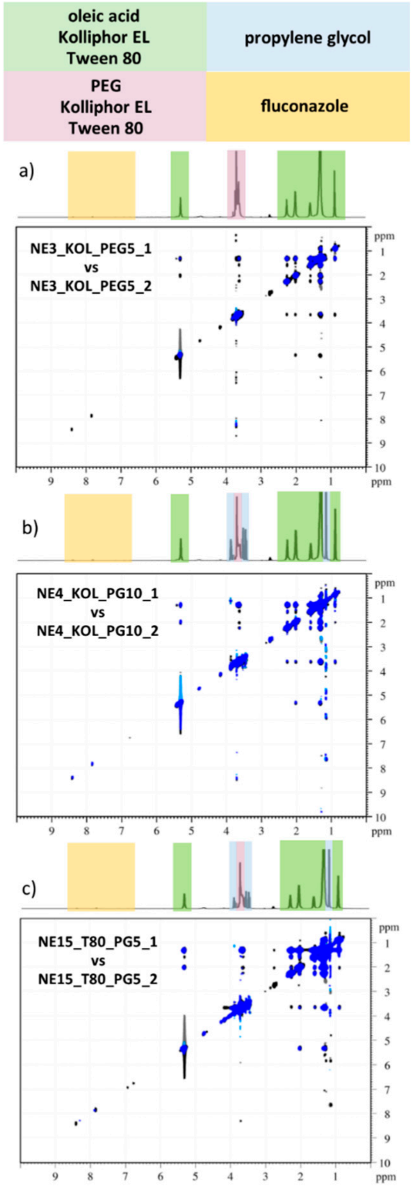

Figure S6. ^{1}H-^{1}H NOESY NMR spectra of NE3_KOL_PEG5, NE4_KOL_PG10, NE15_T80_PG5 (series 1 presented in black and 2 in blue).
